# DNMT3B- SLC25A6 axis-mediated DNA methylation regulates ferroptosis to promote paclitaxel resistance in triple-negative breast cancer

**DOI:** 10.3389/fcell.2026.1887314

**Published:** 2026-07-30

**Authors:** Hao Wu, Tao Zhu, Tianyuan Li, Zhenggui Du

**Affiliations:** 1 Department of General Surgery, West China Hospital, Sichuan University, Chengdu, China; 2 Breast Center, West China Hospital, Sichuan University, Chengdu, China

**Keywords:** DNA Methylation, DNMT3B- SLC25A6 axis, ferroptosis, paclitaxel resistance, triple-negative breast cancer (TNBC)

## Abstract

**Background:**

Triple negative breast cancer (TNBC) is the most malignant type of breast cancer, and its treatment usually uses paclitaxel for chemotherapy. However, TNBC cells are increasingly resistant to paclitaxel. In the context of paclitaxel resistance, we investigated the role of DNA methylation and the DNA methyltransferase DNMT3B methylation regulatory proteins, as well as their interaction with ferroptosis regulator SLC25A6, in regulating paclitaxel resistance and ferroptosis in TNBC.

**Methods:**

We used database integration analysis methods, combined with bioinformatics screening, *in vitro* and *in vivo* cell and animal experimental models to analyze DNA methylation and the effect of DNMT3B on SLC25A6 DNA methylation. We explored the effects of the DNMT3B-SLC25A6 regulatory axis on ferroptosis and paclitaxel resistance using electron microscopy, fluorescent probes, and detection of key biomarkers. Statistical significance was evaluated using Student’s t-test and analysis of variance (ANOVA).

**Results:**

DNMT3B was significantly overexpressed in paclitaxel-resistant TNBC tissues and cell lines, correlating with enhanced proliferation, migration, and ferroptosis. Mechanistically, DNMT3B induced DNA methylation of SLC25A6 through its 252-bp CpG island, inhibited its protein expression, and triggered ferroptosis by disrupting mitochondrial function and redox balance, leading to paclitaxel resistance in TNBC.

**Conclusion:**

In paclitaxel-resistant TNBC, DNMT3B regulates SLC25A6 expression via DNA methylation, and the DNMT3B-SLC25A6 regulatory axis acts as a central regulator of ferroptosis, promoting paclitaxel resistance in TNBC by suppressing ferroptosis via oxidative stress imbalance. These findings provide promising therapeutic targets for the intervention of TNBC paclitaxel resistance.

## Introduction

Triple-negative breast cancer (TNBC) is a distinct subtype of breast cancer characterized by negative immunohistochemical staining for estrogen receptor (ER), progesterone receptor (PR), and human epidermal growth factor receptor 2 (HER2) expression (Yin et al., 2020). It accounts for 10.0%–20.8% of all breast cancer subtypes and is characterized by aggressive biological behavior, high malignancy, and a propensity for recurrence and metastasis, resulting in a relatively poor prognosis (Liu et al., 2023; Bianchini et al., 2022). Chemotherapy is the mainstay of treatment for TNBC, but the emergence of drug resistance is becoming increasingly severe (Zhu et al., 2023).

As a common chemotherapy drug for TNBC, paclitaxel plays an important role in the treatment of TNBC, but its widespread drug resistance poses a serious challenge to the chemotherapy of TNBC (Marra and Curigliano, 2021; Won and Spruck, 2020). The study found that in TNBC, ABCB1 overexpression is an important driving factor of paclitaxel resistance, and such changes affect drug metabolism and clearance (Ou et al., 2025). Activation of the NF-κB signaling pathway significantly enhances EMT in TNBC and promotes resistance to paclitaxel (Das et al., 2025). In addition, the expression of HER2 and β - catenin was significantly upregulated in paclitaxel-resistant cells, promoting the expression of TCF-4, TCF-1, p-GSK3 β, Cyclin D1, and c-Myc, resulting in reduced apoptosis and significantly increased proliferation and growth of TNBC cells (Merikhian et al., 2021).

DNA methylation is an epigenetic modification in which the methyl group of S-adenosylmethionine is transferred to the cytosine C5 site under the catalysis of DNA methyltransferase (Moore et al., 2013). Research has found that multiple resistance-related genes are activated by hypomethylation (Muggerud et al., 2010). For the ABCB1 gene: Hypomethylation in the promoter region leads to overexpression of P-glycoprotein, enhancing drug efflux (Muggerud et al., 2010). In addition, Hypermethylation of the CDH1 promoter and hypomethylation of N-cadherin promotes epithelial–mesenchymal transition (EMT), leading to paclitaxel resistance (Wang et al., 2014). Research shows that in doxorubicin resistant breast cancer, inhibiting the expression of DNA methyltransferase can inhibit the expression of HK2, reverse the global gene inhibition related to histone methyltransferase, and restore chemotherapy sensitivity (Hong and Pan, 2024). DNA methyltransferase inhibitors (dicitabine, 5-azacytidine) combined with paclitaxel can overcome drug resistance by reversing abnormal methylation, degrading DNMT protein, inducing virus mimic reaction and other mechanisms, and provide a new combination scheme for the treatment of breast cancer (Christman, 2002; Stelmach and Trumpp, 2023).

## Materials and methods

### Clinical sample collection

The study was approved by the Institutional Research Ethics Committee of West China Hospital of Sichuan University and conducted according to the principles of the Declaration of Helsinki. Breast cancer samples were obtained from postoperative specimens from patients diagnosed with TNBC. Tumor tissues were embedded in paraffin for immunohistochemistry and frozen at −80 °C for RNA and protein extraction.

### Cell cultures and treatments

The Human TNBC cell lines (Hs578T, MDA-MB-231) were all purchased from the American Type Culture Collection (ATCC) and cultured according to the manufacturer’s instructions. Cells were cultured in a humidified atmosphere containing 5% CO2 at 37 °C. 5-Aza-2 ′- deoxycytidine (HY-A0004) and Ferrostatin-1 (HY-100579) are both from MedChemExpress and the cells were processed according to the instructions.

### RNAi and plasmid transfection

We designed and constructed plasmids for overexpression of DNMT3B and SLC25A6, as well as shRNA constructs for knockdown of these genes. Transfections were performed using Lipofectamine 3,000 according to the manufacturer’s instructions. Stable cell lines were generated via lentiviral packaging and selection with puromycin.

### RNA extraction and reverse transcription-quantitative (RT-qPCR)

Total RNA was extracted from cell lines using TRIzol reagent (Takara Bio). The extracted RNA was reverse transcribed to cDNA with PrimeScript RT Reagent (Takara Bio, Inc.). Real-time qPCR was performed with a Prime-Script ® RT Reagent Kit (Takara Bio, Inc.) and a LightCycler system (Roche Diagnostics) was used for detection. GADPH as normalization control.

### Immunohistochemistry

Tissue sections were rehydrated in xylene and alcohol, and then rehydrated at 37 °C with 3% H_2_O_2_ for 30 min. Next, all slices were incubated with goat serum for 15 min, and then washed with TBST three times. 10 min each time. Then, the sections were incubated overnight with Ki67 (1:1000, Abcam), SLC25A6(1:500, Abcam), FTH1(1: 1000, Abcam), GPX4(1: 1000, Abcam) and NOX1 (1: 1000, Abcam) at 4 °C. Next, the sections were incubated with secondary antibody (HRP rabbit/mouse En Vision) at 37 °C for 30 min. DAB (Boster) was used for visual color rendering of the signal.

### Western blot analysis

Total protein was extracted from TNBC cell and tissue samples using RIPA lysis buffer (Beyotime Institute of Biotechnology). After separation by 10% SDS-PAGE (20 µg protein per lane), the total protein was transferred to PVDF membranes (MilliporeSigma). The PVDF membranes were blocked with 5% nonfat milk powder (Beyotime). The membranes were then incubated overnight at 4 °C with antibodies against DNMT3B (1:1000; Abcam), SLC25A6 (1:5000; Abcam), GPX4 (1:1000; Abcam), GSH (1:500; Abcam), and GAPDH (1:5000; Abcam). Finally, protein expression was analyzed using chemiluminescence reagents (Hyperfilm ECL).

### Mitochondrial electron microscopy

The samples were pre-fixed with 2.5% glutaraldehyde (fixed by the customer) and post-fixed with 1% osmium tetroxide. They were then dehydrated with acetone in a graded series (30% → 50% → 70% → 80% → 90% → 95% → 100%, with three changes of 100% acetone). The dehydrated samples were infiltrated with Epon-812 embedding medium in the following ratios: 3:1, 1:1, and 1:3 (dehydrant to embedding medium). The samples were then embedded in pure Epon-812. Ultrathin sections (60–90 nm) were cut using an ultramicrotome and mounted on copper grids. The sections were stained with uranyl acetate for 10–15 min, followed by lead citrate for 1–2 min at room temperature. Images were captured using a JEM-1400FLASH transmission electron microscope produced by JEOL. Each copper grid was first observed at low magnification to view the entire tissue, and images were captured from the selected areas of interest.

### Malondialdehyde (MDA) assay

Cells were seeded in 6-well plates and treated in the same manner as for the C11-BODIPY assay. After treatment, the cells were collected for protein extraction. The protein concentration was determined using a BCA Protein Assay Kit (Thermo Fisher Scientific, United States), and the MDA concentration was quantitatively measured using a Lipid Peroxidation MDA Assay Kit (Beyotime Biotechnology, China) according to the manufacturer’s instructions. The ratio of MDA to protein concentration was then calculated.

### Intracellular labile iron measurement

The levels of labile iron in TNBC cells were assessed using the Fe^2+^ -specific fluorescent probe FerroOrange (MKBio, China). Cells were cultured in 6-well plates and pre-treated with doxorubicin for 48 h. Subsequently, the cells were washed and incubated with 1 μmol/L FerroOrange for 30 min to obtain the fluorescence intensity.

### Lipid Peroxidation Assay

To measure lipid peroxidation levels as an indicator of ferroptosis toxicity, the Image-iT™ Lipid Peroxidation Kit (ThermoFisher) was used according to the manufacturer’s protocol. Cells stained with the BODIPY™581/591 C^11^ dye provided in the kit for 30 min were analyzed by flow cytometry. The signal shift from red to green that occurs with lipid oxidation was quantified by taking the average fluorescence emission ratio of approximately 590 nm to approximately 510 nm to measure hydroperoxides and plotted as relative ferroptosis levels.

### Data filtering and mapping

The following websites and software were used to process and visualize the data: The Human Protein Atlas (https://www.proteinatlas.org/, UCSC (http://www.genome.ucsc.edu), Methprimer (http://www.urogene.org/cgi-bin/methprimer/methprimer.cgi), EBI CpGplot (http://www.ebi.ac.uk/Tools/seqstats/emboss_cpgplot/), National Center for Biotechnology Information database (https://www.ncbi.nlm.nih.gov/) and Genomic tRNA Database (GtRNAdb, http://gtrnadb.ucsc.edu/).

### Cellular immunofluorescence

TNBC cells were seeded on coverslips and cultured. Subsequently, the slides containing the cells were fixed with 4% paraformaldehyde at room temperature for 30 min and permeabilized with 0.25% Triton X-100 solution for 20 min. The cell slides were washed three times with PBS for 5 min each, and then blocked with 5% bovine serum albumin at room temperature for 1 h. The coverslips were incubated with primary antibodies against GPX4 (1:500, Abcam, United States), COX2 (1:800, Abcam, United States), FTH1 (1:500, Abcam, United States), and NOX1 (1:1,000, Abcam, United States) at 4 °C overnight. After washing three times with PBS for 5 min each, the coverslips were incubated with secondary antibodies and DAPI.

### Cell counting Kit-8 (CCK-8) assay

TNBC cells were transfected and then seeded into 96-well plates (2 × 10^3^ cells per well). After incubation, CCK-8 assay reagent (Dojindo Molecular Technologies, Inc.) was added to each well containing cells, and the plate was incubated. The absorbance at 450 nm was recorded for each well to analyze cell proliferation.

### Lipid ROS measurement

Lipid ROS were detected using the BODIPY 581/591 C^11^ probe (Thermo Fisher Scientific, D3861) according to the manufacturer’s instructions. Briefly, cells were incubated with a final concentration of 5 µM BODIPY 581/591 C^11^ at 37 °C for 30 min and then washed three times with PBS. Oxidation of the polyunsaturated butadiene moiety of the dye causes a shift in fluorescence emission from 590 nm to 510 nm, which was measured using a BD Accuri C6 Plus flow cytometer (BD Biosciences).

### Mouse xenograft tumor model

All animal experiments were conducted in accordance with institutional guidelines and approved by the Animal Experiment Ethics Committee of West China Hospital, Sichuan University. Female BALB/c-nu mice were purchased from Chengdu Dasuo Experimental Animal Co., Ltd (Chengdu, China) and housed in the Experimental Animal Center of West China Hospital, Sichuan University. Thirty-six hours after transfection, TNBC cells (5 × 10^6^) were subcutaneously injected into the mammary fat pads of female athymic nude mice. Tumor volume was calculated according to the following formula: Volume = (width^2^ x length)/2.

### FeRhoNox-1 assay

Remove FeRhoNox-1 (MX4558, MKBio) from −20 °C and let it sit at room temperature for 30 min, followed by low-speed centrifugation to precipitate the powder. Add 109 μL of DMSO to 50 μg of the powder, and pipette up and down more than 5 times to dissolve it completely. Dilute the stock solution with phenol red-free HBSS or PBS. Aspirate the culture medium from the cell culture dish and gently wash it twice with PBS. Add the working solution containing 5 μM FeRhoNox-1, and incubate at 37 °C with 5% CO_2_ for 60 min. After incubation, gently wash the cells three times with PBS to remove unbound probes. Observe the expression of Fe^2+^ under a fluorescence microscope.

### Cell migration and invasion assays

In brief, treated cells were seeded into the upper chamber of a Transwell insert. Subsequently, 550 µL of high-glucose DMEM containing 10% FBS was added to the corresponding lower chamber. After 48 h of incubation, MDA-MB-231 and Hs578T cells migrated to the lower chamber. For the invasion assay, the insert was pre-coated with Matrigel (1 mg/mL). Cells that penetrated to the lower chamber were fixed with methanol for 20 min, and their outlines were stained with 0.1% crystal violet.

### Wound healing assay

5 × 10^5^ MDA-MB-231 and Hs578T cells were grown in six-well plates. After reaching confluence, non-adherent cells were washed away twice with phosphatebuffered saline (PBS). The cell monolayer was scratched with a pipette tip (10 mL) to generate 3 scratch wounds and then rinsed twice with PBS to remove non-adherent cells. After 0, and 48 h, the distance between the wound sides was measured.

### Cell apoptosis

MDA-MB-231 and Hs578T cells apoptosis were quantified using a FITC-labeled Annexin V/propidium iodide (PI) Apoptosis Detection kit (Beyotime, China) according to the instructions. Flow cytometric analysis was performed immediately after staining using a flow cytometer (Beckman, United States).

### Colony-forming assay

For the colony-forming assay, MDA-MB-231 and Hs578T cells were plated in 6-well plates (500 cells/well) containing 2.5 mL of medium and cultured for 3 weeks. Colonies formed by cell proliferation were stained with crystal violet, and colonies containing at least 50 cells were counted. Colonies were fixed with 20% methanol and stained with 0.1% crystal violet, and the colonies were counted.

### Bisulfite sanger sequencing

At least 500 ng of genomic DNA extracted from TNBC patient specimens, MDA-MB-231 and Hs578T cells were bisulfite converted using a MethylCode™ Bisulfite Conversion Kit (Applied Biosystems, United States). The SLC25A6 promoter was amplified by PCR with Taq DNA Polymerase (Invitrogen, United States). The primer sequence was designed using Methyl Primer Express™ Software v1.0 (Applied Biosystems, United States). The PCR products were electrophoresed, purified using Spin-X tubes, and then cloned into the pUC-T vector (both from CWbiotech, Beijing, China). Ten single products were sequenced for each sample.

### Statistical analysis

GraphPad Prism (version 8.4.3) was used for data collection and analysis in this study. Typically, the Student’s unpaired t-test was employed to determine the statistical significance effect between two groups of data; one-way or two-way analysis of variance (ANOVA) was used to determine the statistical significance effect among multiple groups of data. The significance levels were indicated by the following footnotes: *p < 0.05, **p < 0.01, ***p < 0.001.

## Results

### DNMT3B is significantly overexpressed in paclitaxel resistant TNBC

We established paclitaxel-resistant strains of MDA-MB-231 (MDA-MB-231-PTXS) and Hs578T (Hs578T-PTXS), and functional cell assays revealed that the proliferation and colony-forming abilities of these resistant strains were significantly increased compared to normal cell lines (Supplementary Figure S1A,B). Additionally, their apoptotic capacity was markedly reduced (Supplementary Figure S1C). We also observed that the migration and invasion abilities of MDA-MB-231-PTXS and Hs578T-PTXS were significantly enhanced compared to parental cell lines. (Supplementary Figure S1D).

**FIGURE 1:**
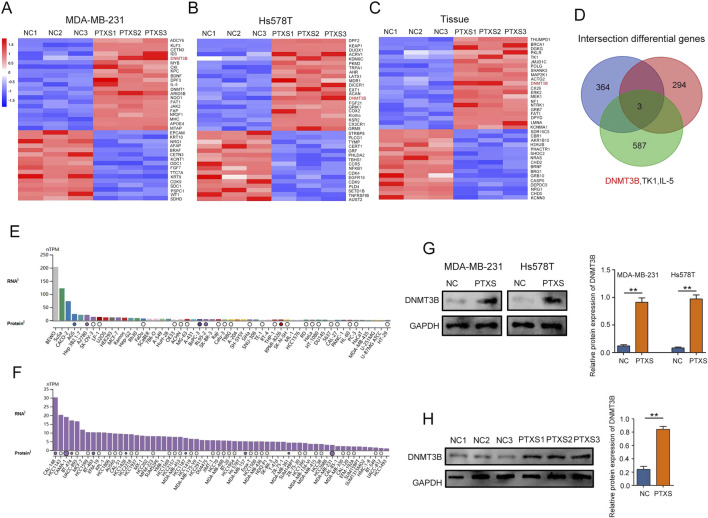
DNMT3B is significantly overexpressed in TNBC paclitaxel resistant. **(A)** Sequencing analysis of differential gene expression between MDA-MB-231-PTXS and normal cell lines. **(B)** Sequencing analysis of differential gene expression between Hs578T-PTXS and normal cell lines. **(C)** Sequencing analysis of differential gene expression between TNBC paclitaxel resistant and non-resistant tissues in clinical samples. **(D)** Wayne diagram analysis of sequencing results of three sets of samples. **(E,F)** Analysis of DNMT3B expression in tumor cell lines and breast cancer cell lines by biological database. **(G)** WB analysis of DNMT3B expression in MDA-MB-231-PTXS and Hs578T-PTXS. **(H)** WB analysis of DNMT3B expression in TNBC paclitaxel resistant and non-resistant tissues in clinical samples. **p < 0.01.

High-throughput sequencing of MDA-MB-231-PTXS, Hs578T-PTXS and human TNBC paclitaxel-resistant samples, we found that DNMT3B was highly expressed in TNBC paclitaxel-resistant samples (Figures 1A–D). Additionally, we found through bioinformatic databases that DNMT3B is lowly expressed in breast cancer cell lines (Figures 1E,F). Furthermore, we found that DNMT3B protein expression was significantly increased in MDA-MB-231-PTXS and Hs578T-PTXS (Figure 1G). DNMT3B was also found to be significantly increased in paclitaxel-resistant TNBC clinical samples (Figure 1H).

### DNMT3B regulates the malignant biological behavior through ferroptosis in TNBC paclitaxel resistant strains

Studies have found that ferroptosis plays an important role in the malignant progression of TNBC and chemotherapeutic drug resistance, while drugs targeting DNA methylation (such as decitabine and azacitidine) are also widely used in the chemotherapy of TNBC (Wang et al., 2015; Mokhtarpour et al., 2024). So, do DNA methylation and ferroptosis jointly play an important role in the malignant progression of paclitaxel-resistant TNBC?

**FIGURE 2:**
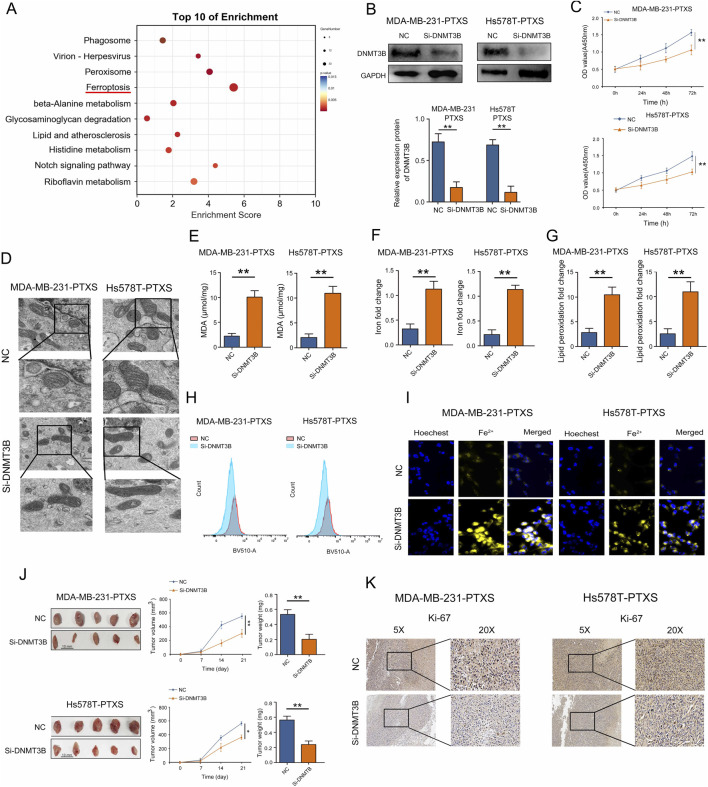
DNMT3B regulates the malignant biological behavior through ferroptosis in TNBC paclitaxel resistant strains. **(A)** Cluster analysis of differential genes involving signaling pathways. **(B)** WB analysis of the expression of DNMT3B in MDA-MB-231-PTXS and Hs578T-PTXS. **(C)** Effect of DNMT3B on proliferation in MDA-MB-231-PTXS and Hs578T-PTXS. **(D)** Electron microscopy analysis of the effect of DNMT3B on mitochondrial morphology in MDA-MB-231-PTXS and Hs578T-PTXS. **(E)** MDA assay analysis of the effect of DNMT3B on malondialdehyde in MDA-MB-231-PTXS and Hs578T-PTXS. **(F)** Iron ion assay analysis of the effect of DNMT3B on iron ion in MDA-MB-231-PTXS and Hs578T-PTXS. **(G)** Lipid Peroxidation Assay analysis of the effect of DNMT3B on lipid peroxidation in MDA-MB-231-PTXS and Hs578T-PTXS. **(H)** Lipid ROS assay analysis of the effect of DNMT3B on ROS in MDA-MB-231-PTXS and Hs578T-PTXS. **(I)** Immunofluorescence analysis of the effect of DNMT3B on Fe^2+^ in MDA-MB-231-PTXS and Hs578T-PTXS. **(J)** *In vivo* animal model analysis of tumorigenesis in MDA-MB-231-PTXS and Hs578T-PTXS. **(K)** Immunohistochemical analysis of the expression of Ki-67 in MDA-MB-231-PTXS and Hs578T-PTXS. *p < 0.05. **p < 0.01.

First, we performed clustering analysis on the previous sequencing results and found that ferroptosis was a significantly correlated signaling pathway in paclitaxel-resistant TNBC cell lines (Figure 2A). Electron microscopy revealed that the mitochondrial membrane density of paclitaxel-resistant TNBC cells was more normal, with a significant reduction in cellular ferroptosis (Supplementary Figure S2A). The levels of MDA, Fe^2+^, and lipid peroxidation were significantly decreased in paclitaxel-resistant TNBC cells (Supplementary Figure S2B-D). ROS and Fe^2+^ were also significantly reduced in paclitaxel-resistant TNBC cells (Supplementary Figure S2E,F). In addition, the addition of Ferrostatin-1 further confirms the significant reduction of ferroptosis in MDA-MB-231-PTXS, Hs578T-PTXS (Supplementary Figure S3). Therefore, we knocked down DNMT3B expression in paclitaxel-resistant TNBC cells and found that cell proliferation was significantly inhibited (Figures 2B,C). Electron microscopy showed that reducing DNMT3B expression induced mitochondrial shrinkage and increased membrane density, with a significant increase in ferroptosis (Figure 2D). Reducing DNMT3B expression also elevated the levels of MDA, Fe^2+^, and lipid peroxidation (Figures 2E–G). ROS levels also increased with the reduction of DNMT3B expression (Figure 2H). Reducing DNMT3B expression elevated mitochondrial superoxide Fe^2+^ levels (Figure 2I). In addition, reducing DNMT3B expression significantly impaired TNBC cell proliferation *in vitro* (Figure 2J) and suppressed Ki-67 expression in mouse xenografts (Figure 2K). The above results indicate that DNMT3B significantly affects ferroptosis in paclitaxel-resistant TNBC.

### DNMT3B inhibits SLC25A6 expression by promoting hypermethylation of the CpG island in the SLC25A6 promoter

**FIGURE 3:**
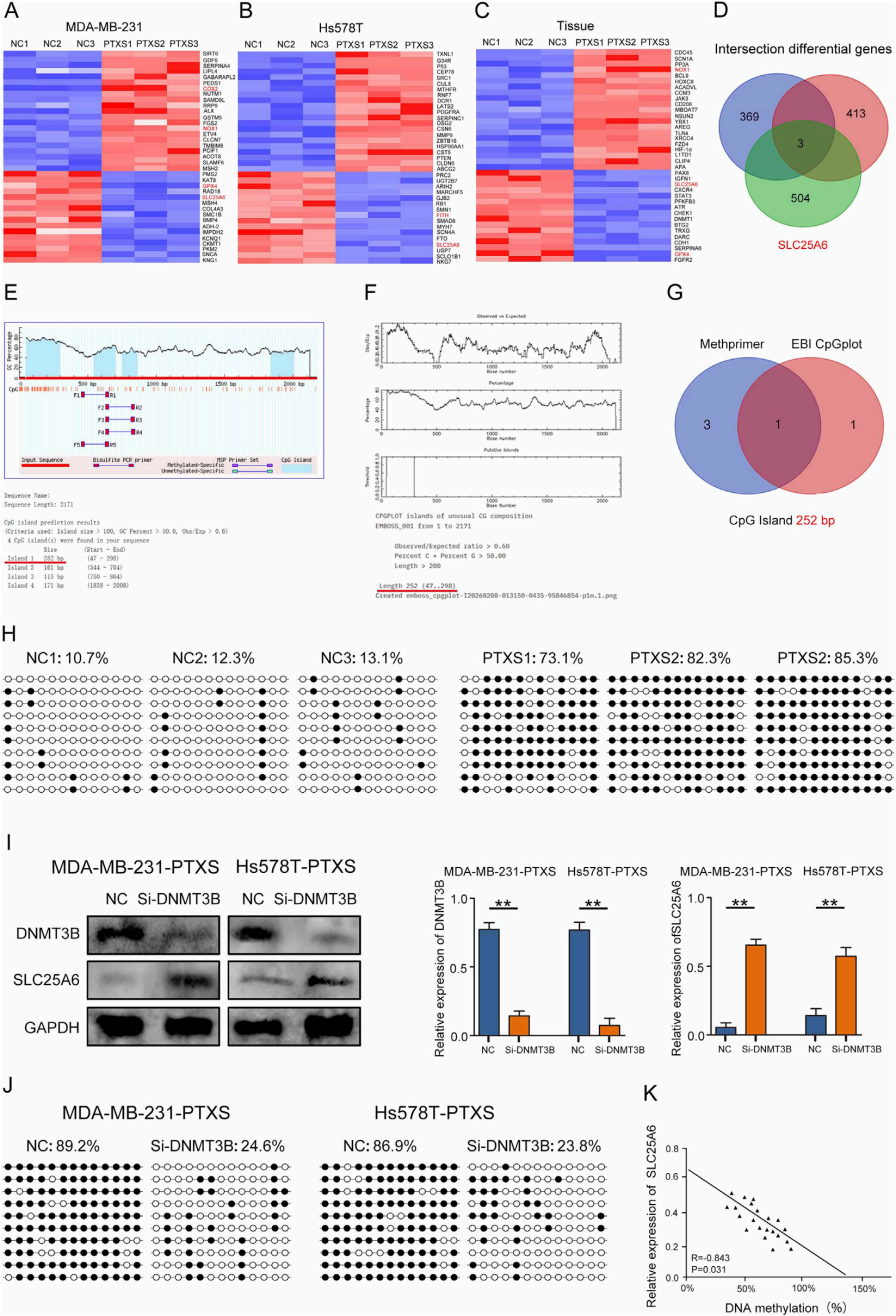
DNMT3B inhibits SLC25A6 expression by promoting hypermethylation of the CpG island in the SLC25A6 promoter. **(A)** Sequencing analysis of ferroptosis related differentially expressed genes in paclitaxel resistant MDA-MB-231. **(B)** Sequencing analysis of ferroptosis related differentially expressed genes in paclitaxel resistant Hs578T. **(C)** Sequencing analysis of ferroptosis related differentially expressed genes between TNBC paclitaxel resistant and non-resistant tissues in clinical samples. **(D)** Wayne diagram analysis of sequencing results of three sets of samples. **(E,F)** Bioinformatics database shows potential DNA methylation CpG island sites in SLC25A6. **(G)** Wayne diagram analysis of potential DNA methylation CpG island sites in SLC25A6 of two database datas. **(H)** Bisulfite sequencing analysis was performed on SLC25A6 252-bp CpG island methylation between TNBC paclitaxel resistant and non-resistant tissues in clinical samples. **(I)** WB analysis of the expression of DNMT3B and SLC25A6 in paclitaxel resistant MDA-MB-231 and Hs578T cells. **(J)** Bisulfite sequencing analysis of the effect of DNMT3B on SLC25A6 252-bp CpG island methylation in MDA-MB-231-PTXS and Hs578T-PTXS. **(K)** Statistical analysis of the association between DNA methylation of the 252-bp CpG island in the SLC25A6 and its expression. **p < 0.01.

DNMT3B plays a major role in the *de novo* methylation of genes (Li et al., 2016; Man et al., 2022). We sought to further investigate the underlying molecular mechanisms by which DNMT3B exerts its functional effects on paclitaxel-resistant TNBC. We first analyzed the expression of ferroptosis-related proteins in MDA-MB-231-PTXS, Hs578T-PTXS and human TNBC paclitaxel-resistant samples, and found that SLC25A6 was low expressed (Figures 3A–D). Importantly, through the DNA methylation profile on the DNA methylation bioinformatics website, it was discovered that SLC25A6 has a potential DNA methylation CpG island of 252-bp (Figures 3F,G). More important, we verified that SLC25A6 exhibits hypermethylation at 252-bp CpG island in TNBC paclitaxel-resistant samples (Figure 3H). Furthermore, Western blot results showed that reducing DNMT3B expression increased SLC25A6 expression (Figure 3I). Additionally, in MDA-MB-231-PTXS, Hs578T-PTXS, inhibiting the expression of DNMT3B significantly reduces the DNA methylation of SLC25A6 at 252-bp CpG island, and there was a negative correlation between DNA methylation and SLC25A6 expression (Figures 3J,K). In addition, 5-Aza-2 ′- deoxycytidine (a DNA methyltransferase inhibitor) significantly inhibited proliferation and ferroptosis in MDA-MB-231-PTXS, Hs578T-PTXS (Supplementary Figure S4). The above results collectively confirm that DNMT3B significantly regulates the expression of SLC25A6 and ferroptosis of MDA-MB-231-PTXS, Hs578T-PTXS through DNA methylation.

### Increased SLC25A6 inhibits malignant biological behaviors of paclitaxel-resistant TNBC through ferroptosis

**FIGURE 4:**
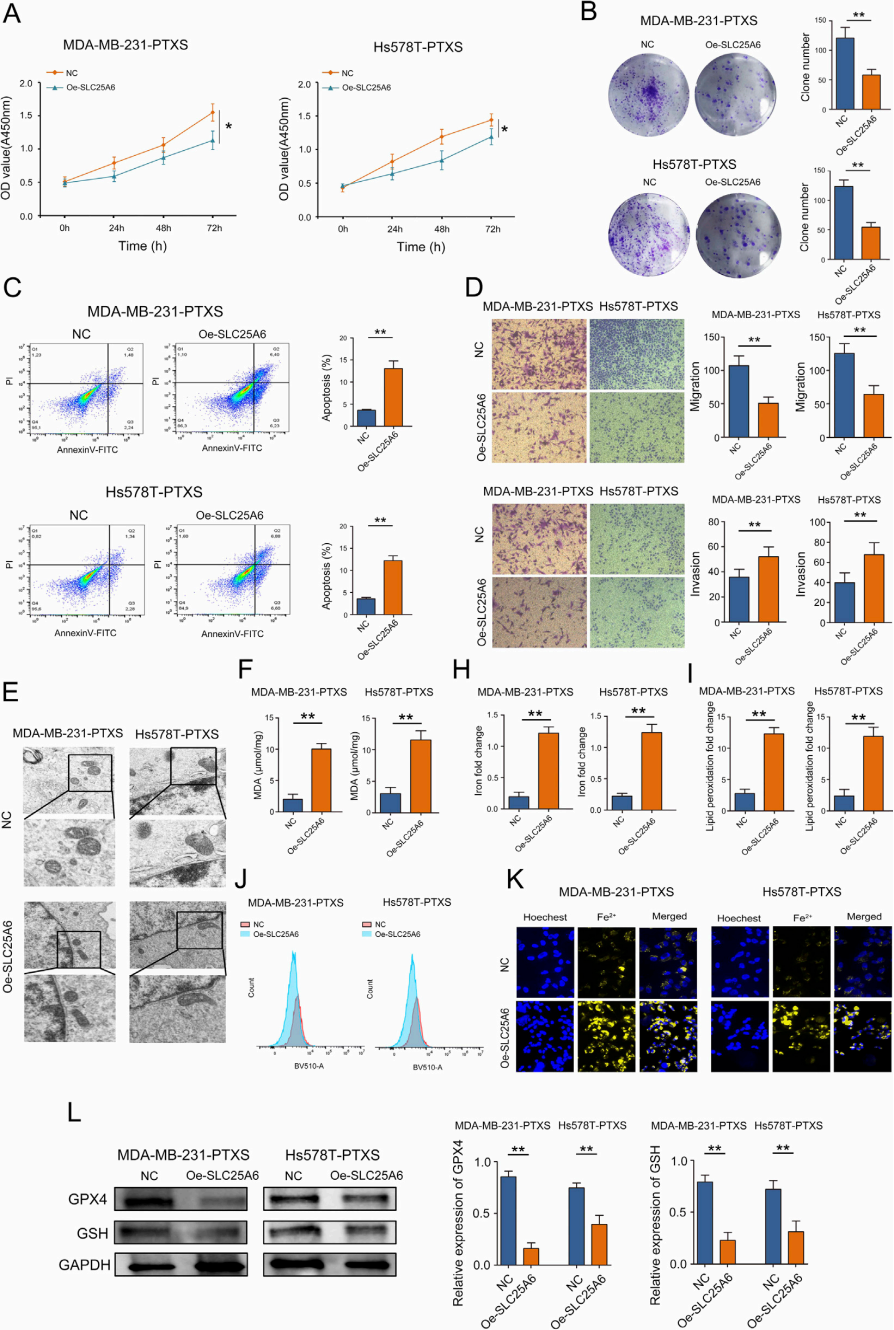
Increased SLC25A6 inhibits malignant biological behaviors of paclitaxel-resistant TNBC through ferroptosis. **(A)** Effect of SLC25A6 on proliferation in MDA-MB-231-PTXS and Hs578T-PTXS. **(B)** Effect of SLC25A6 on clone formation in MDA-MB-231-PTXS and Hs578T-PTXS. **(C)** Effect of SLC25A6 on apoptosis in MDA-MB-231-PTXS and Hs578T-PTXS. **(D)** Effect of SLC25A6 on invasion and migration in MDA-MB-231-PTXS and Hs578T-PTXS. **(E)** Electron microscopy analysis of the effect of SLC25A6 on mitochondrial morphology in MDA-MB-231-PTXS and Hs578T-PTXS. **(F)** MDA assay analysis of the effect of SLC25A6 on malondialdehyde in MDA-MB-231-PTXS and Hs578T-PTXS. **(H)** Iron ion assay analysis of the effect of SLC25A6 on iron ion in MDA-MB-231-PTXS and Hs578T-PTXS. **(I)** Lipid Peroxidation Assay analysis of the effect of SLC25A6 on lipid peroxidation in MDA-MB-231-PTXS and Hs578T-PTXS. **(J)** Lipid ROS assay analysis of the effect of SLC25A6 on ROS in MDA-MB-231-PTXS and Hs578T-PTXS. **(K)** Immunofluorescence analysis of the effect of SLC25A6 on Fe^2+^ in MDA-MB-231-PTXS and Hs578T-PTXS **(L)** WB analysis of the effect of SLC25A6 on expression of GPX4 and GSH in MDA-MB-231-PTXS and Hs578T-PTXS. **p < 0.01.

To further investigate the effects of SLC25A6 on the malignant biological behaviors of paclitaxel-resistant TNBC, gain-of-function experiments were conducted in paclitaxel-resistant TNBC cells. As shown by CCK-8 assays, elevated SLC25A6 expression markedly suppressed cell proliferation in MDA-MB-231-PTXS and Hs578T-PTXS (Figure 4A). These findings were further corroborated by colony formation experiments (Figure 4B). Fluorescein isothiocyanate (FITC)-conjugated Annexin V and PI staining was then used to measure the effect of SLC25A6 on apoptosis. The results indicate that elevated SLC25A6 significantly induced apoptosis in MDA-MB-231-PTXS and Hs578T-PTXS (Figure 4C). Transwell assays demonstrated that elevated SLC25A6 significantly suppressed the invasion and migration abilities in MDA-MB-231-PTXS and Hs578T-PTXS (Figure 4D). More importantly, we found that elevated SLC25A6 caused mitochondrial shrinkage and increased membrane density in MDA-MB-231-PTXS and Hs578T-PTXS, with a significant increase in ferroptosis (Figure 4E). Increasing SLC25A6 expression also elevated the levels of MDA, Fe^2+^, and lipid peroxidation (Figures 4F,H,I). ROS were also elevated concomitant with increased SLC25A6 expression (Figure 4J). Elevated SLC25A6 expression markedly enhanced the levels of mitochondrial superoxide Fe^2+^(Figure 4K). WB results also confirmed that GPX4 and GSH were significantly reduced (Figure 4L).

### DNMT3B regulates the expression of SLC25A6 to affect the malignant biological behavior of paclitaxel-resistant TNBC through ferroptosis

**FIGURE 5:**
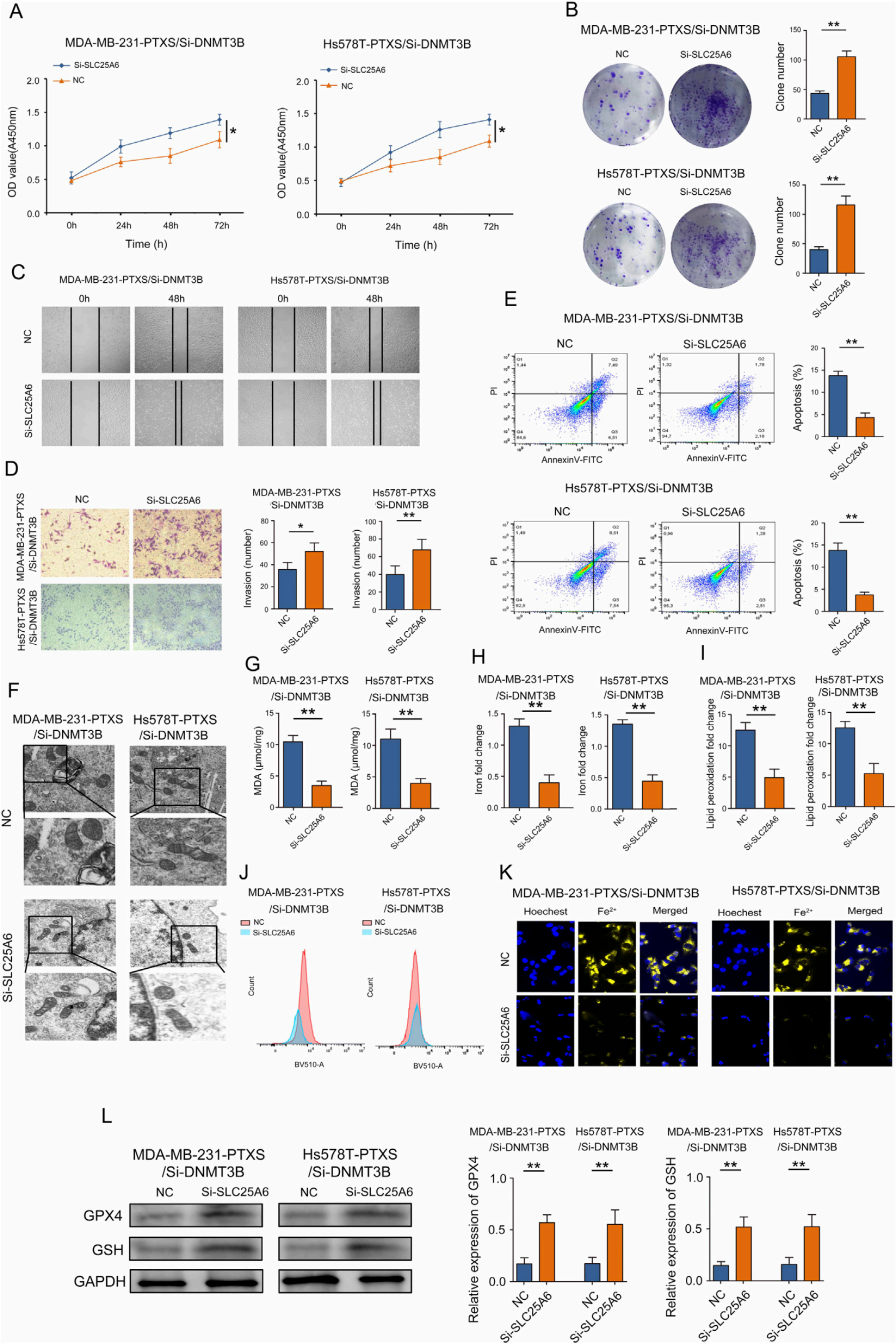
DNMT3B regulates the expression of SLC25A6 to affect the malignant biological behavior of paclitaxel-resistant TNBC through ferroptosis. **(A)** CCK8 analysis of cell proliferation in paclitaxel-resistant TNBC/Si-DNMT3B after down-expression of SLC25A6. **(B)** Clone formation in paclitaxel-resistant TNBC/Si-DNMT3B after down-expression of SLC25A6. **(C)** Cell migration in paclitaxel-resistant TNBC/Si-DNMT3B after down-expression of SLC25A6. **(D)** Cell invasion in paclitaxel-resistant TNBC/Si-DNMT3B after down-expression of SLC25A6. **(E)** Cell apoptosis in paclitaxel-resistant TNBC/Si-DNMT3B after down-expression of SLC25A6. **(F)** Electron microscopy analysis of mitochondrial morphology in paclitaxel-resistant TNBC/Si-DNMT3B after down-expression of SLC25A6. **(G)** MDA assay analysis of malondialdehyde in paclitaxel-resistant TNBC/Si-DNMT3B after down-expression of SLC25A6. **(H)** Iron ion assay analysis of iron ion in paclitaxel-resistant TNBC/Si-DNMT3B after down-expression of SLC25A6. **(I)** Lipid peroxidation assay analysis of lipid peroxidation in paclitaxel-resistant TNBC/Si-DNMT3B after down-expression of SLC25A6. **(J)** Lipid ROS assay analysis of ROS in paclitaxel-resistant TNBC/Si-DNMT3B after down-expression of SLC25A6. **(K)** Immunofluorescence analysis of Fe^2+^ in paclitaxel-resistant TNBC/Si-DNMT3B after down-expression of SLC25A6 **(L)** WB analysis of the effect of SLC25A6 on expression of GPX4 and GSH in paclitaxel-resistant TNBC/Si-DNMT3B after down-expression of SLC25A6. *p < 0.05, **p < 0.01.

Furthermore, we inhibited the expression of SLC25A6 in paclitaxel resistant TNBC with low expression of DNMT3B, and found that the inhibited proliferation ability was restored (Figure 5A). These findings were further validated by cell colony formation experiments (Figure 5B). The inhibited cell migration and invasion also recover with the decrease of SLC25A6 expression in MDA-MB-231-PTXS and Hs578T-PTXS with low expression of DNMT3B (Figures 5C,D). In addition, reducing the expression of SLC25A6 significantly inhibited cell apoptosis in MDA-MB-231-PTXS and Hs578T-PTXS with low expression of DNMT3B (Figure 5E). In the effect on ferroptosis, reducing SLC25A6 leads to mitochondrial contraction and decreased membrane density in MDA-MB-231-PTXS and Hs578T-PTXS with low expression of DNMT3B (Figure 5F). Inhibition of SLC25A6 expression significantly suppressed the levels of mitochondrial superoxide Fe^2+^in MDA-MB-231-PTXS and Hs578T-PTXS with low expression of DNMT3B (Figures 5G–I). In addition, ROS increases with the inhibition of SLC25A6 (Figure 5J). Inhibition of SLC25A6 expression significantly reduced the levels of mitochondrial superoxide Fe^2+^ levels (Figure 5K). WB results also confirmed that the expression of GPX4 and GSH was partially restored (Figure 5L). More importantly, the recovery experiment of overexpression of SLC25A6 in DNMT3B knockdown cells further confirmed that DNMT3B affects the ferroptosis and proliferation by regulating the expression of SLC25A6 in MDA-MB-231-PTXS and Hs578T-PTXS (Supplementary Figure S5).

**FIGURE 6:**
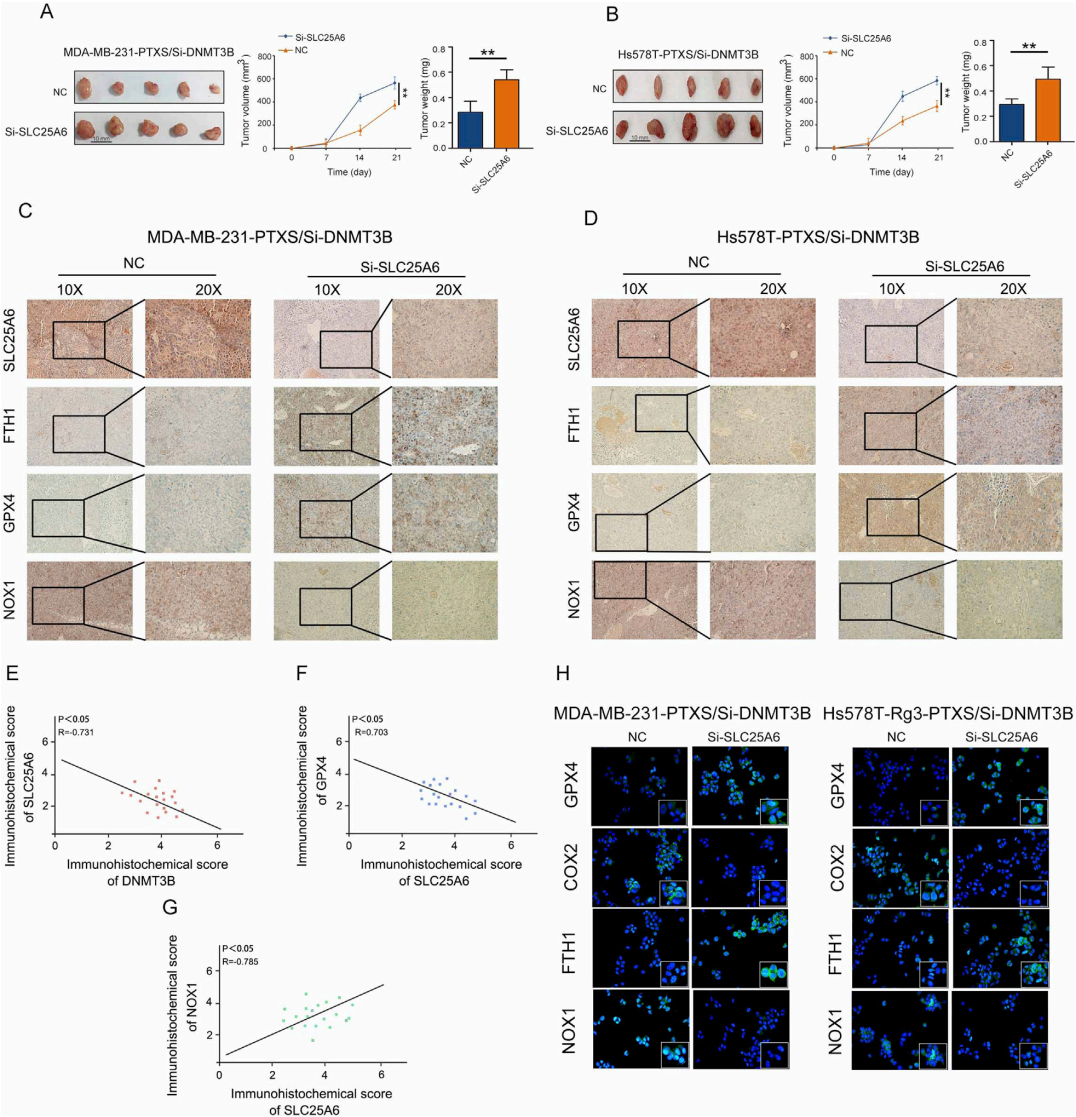
DNMT3B regulates the expression of SLC25A6 to affect ferroptosis in paclitaxel-resistant TNBC. **(A)** *In vivo* animal model analysis of tumorigenesis in paclitaxel-resistant MDA-MB-231/Si-DNMT3B after down-expression of SLC25A6. **(B)** *In vivo* animal model analysis of tumorigenesis in paclitaxel-resistant Hs578T/Si-DNMT3B after down-expression of SLC25A6. **(C)** Immunohistochemical analysis of the expression of key ferroptosis proteins in paclitaxel-resistant MDA-MB-231-PTXS/Si-DNMT3B after down-expression of SLC25A6. **(D)** Immunohistochemical analysis of the expression of key ferroptosis proteins in paclitaxel-resistant Hs578T-PTXS/Si-DNMT3B after down-expression of SLC25A6. **(E)** Statistical analysis of the relationship between SLC25A6 and DNMT3B. **(F)** Statistical analysis of the relationship between SLC25A6 and GPX4. **(G)** Statistical analysis of the relationship between SLC25A6 and NOX1. **(H)** Immunofluorometric analysis of the expression of key ferroptosis proteins in paclitaxel-resistant TNBC/Si-DNMT3B after down-expression of SLC25A6. **p < 0.01.

**FIGURE 7:**
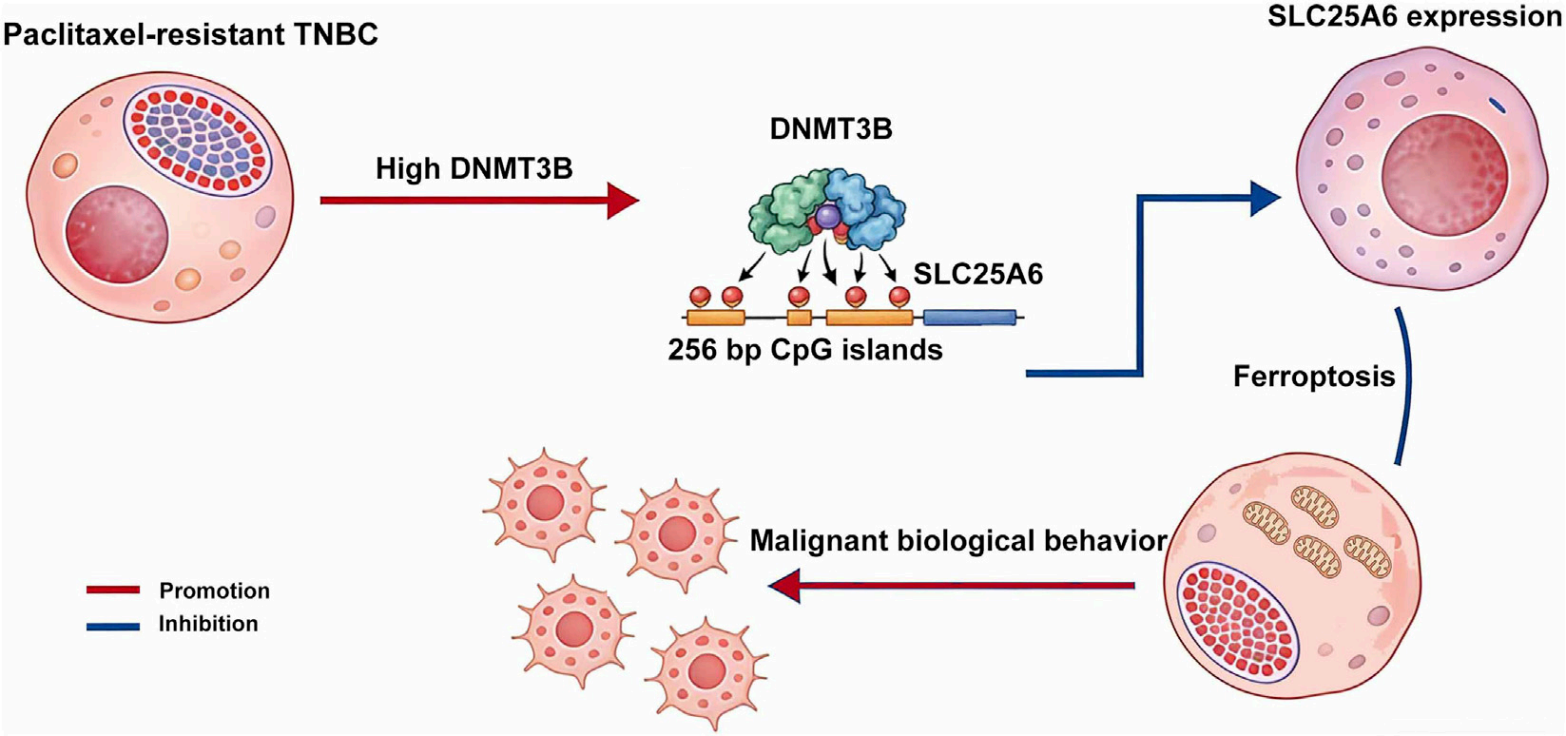
Mechanism diagram: DNMT3B regulates the expression of SLC25A6 through DNA methylation, and the DNMT3B-SLC25A6 regulatory axis acts as a central regulator of iron deposition, inhibiting iron deposition through oxidative stress imbalance and promoting TNBC resistance to paclitaxel.

Furthermore, we analyzed the mechanism underlying DNMT3B-mediated regulation of ferroptosis in paclitaxel resistant TNBC through SLC25A6. *In vivo* animal experiments demonstrated that inhibition of SLC25A6 expression significantly enhanced the tumorigenicity in MDA-MB-231-PTXS and Hs578T-PTXS with low expression of DNMT3B (Figures 6A,B). Furthermore, we analyzed the expression of ferroptosis related proteins and immunohistochemistry confirmed that reducing the expression of SLC25A6 led to an increase in the expression of FTH1 and GPX4, while reducing the expression of NOX1 (Figures 6C,D). Statistical analysis revealed a negative correlation between DNMT3B and SLC25A6 expression, as well as between SLC25A6 and GPX4 expression, and a positive correlation was observed between SLC25A6 and NOX1 expression (Figures 6E–G). More importantly, the results of cellular immunofluorescence also confirmed that reducing the expression of SLC25A6 led to an increase in the expression of FTH1 and GPX4, while reducing the expression of NOX1 (Figure 6H). In summary, our findings indicate that DNMT3B facilitates the malignant biological behavior of paclitaxel-resistant TNBC through DNA methylation at SLC25A6 at 252-bp CpG island, resulting in downregulation of SLC25A6 protein expression and ferroptosis inhibition (Figure 7).

## Discussion

TNBC is a distinct subtype of breast cancer, accounting for approximately 10%–20% of all breast cancer cases. Although TNBC represents a relatively small proportion of breast cancer patients, it poses significant clinical challenges due to its highly aggressive nature, rapid growth rate, and propensity for early metastasis. TNBC patients often present with larger tumor volumes at diagnosis, and the tumors are predominantly high-grade (Grade 3) (So et al., 2022; Li M. et al., 2022). The frequent occurrence of visceral metastases to organs such as the lungs, brain, and liver imposes substantial burdens on both patients and society (Deepak et al., 2020). The standard treatment for TNBC is chemotherapy (Ferrari et al., 2022). However, an increasing number of patients have developed resistance to paclitaxel, a commonly used chemotherapeutic agent (Meng et al., 2025). Therefore, there is an urgent need to elucidate the mechanisms underlying paclitaxel resistance. This study aims to clarify the potential regulatory role of DNA methylation in paclitaxel resistance in TNBC and to investigate the function of ferroptosis in the malignant biological behavior of paclitaxel-resistant TNBC cells. In this study, we found that DNMT3B is highly expressed in paclitaxel-resistant TNBC cells and that DNMT3B regulates SLC25A6 protein expression through DNA methylation. Subsequently, this regulatory mechanism influences malignant progression by modulating ferroptosis in paclitaxel-resistant TNBC cells.

DNA methylation refers to a chemical modification process in which methyl groups are added to specific bases of DNA molecules under the catalysis of DNA methyltransferases (Kulis and Esteller, 2010; Qureshi et al., 2022). It is the most critical chemical modification in epigenetics (Dawson and Kouzarides, 2012). This process of adding methyl groups to DNA bases regulates gene function without altering the DNA sequence (Smith et al., 2025). DNA methyltransferases are a class of key enzymes that catalyze DNA methylation modifications, transferring methyl groups (-CH_3_) from S-adenosylmethionine (SAM) to the C5 position of cytosine, serving as the “executors” of epigenetic regulation (Li Y. et al., 2022). DNMT3B is one of the three major DNA methyltransferases in mammals, belonging to the *de novo* methyltransferase family (Tóth et al., 2025). It is responsible for establishing new methylation marks at unmethylated DNA sites and can completely methylate gene promoter regions (Rolls et al., 2024). DNMT3B is highly expressed in various malignant tumors and significantly promotes their malignant progression (Huang et al., 2023). In hepatocellular carcinoma, DNMT3B is regulated by miR-29c-3p, which affects the DNA methylation level of LATS1 and subsequently modulates the malignant progression of hepatocellular carcinoma through the Hippo signaling pathway (Wu et al., 2019). In addition, DNMT3B can downregulate HOPX expression through DNA methylation and promote the proliferation, migration, and invasion of lung cancer cells, as demonstrated in an *in vivo* xenograft model (Guan et al., 2025). In *Helicobacter* pylori-infected gastric tissues, DNMT3B induces DNA methylation of ID4 (Luan et al., 2025). The hypermethylated status of the ID4 promoter regulates gastric cancer progression through the DEC1/SHH/GLI1 axis (Luan et al., 2025). Currently, demethylating agents targeting DNMT3B, such as decitabine, have been safely used in humans and can be administered as monotherapy or in combination with chemotherapy (Riyas et al., 2025). In this work, we demonstrated that DNMT3B is highly expressed in paclitaxel-resistant TNBC, leading to decreased expression of SLC25A6 through DNA methylation of a 252 bp CpG island. However, in breast cancer data, DNMT3B and SLC25A6 showed a significant positive correlation, where higher DNMT3B expression was associated with higher SLC25A6 expression. High DNMT3B expression may promote the nuclear translocation of transcription-related factors through the Hippo signaling pathway, the BAX/BCL-2 signaling pathway, and other mechanisms, thereby enhancing SLC25A6 expression (Wu et al., 2019). This appears to contradict our research findings. Yet, it is precisely this contradiction that highlights the significance of our study. First, our research revealed that in paclitaxel-resistant breast cancer, high DNMT3B expression leads to increased DNA methylation of SLC25A6, thereby reducing SLC25A6 expression levels. Paclitaxel-resistant breast cancer cells exhibit markedly different biological characteristics compared to their sensitive counterparts, including enhanced proliferative capacity, reduced apoptosis, decreased ferroptosis, and stronger immune evasion capabilities (Dawson and Kouzarides, 2012). These alterations in biological features must be accompanied by changes in gene expression. Our discovery elucidates, from an epigenetic perspective, the mechanism underlying the decreased ferroptosis capacity in paclitaxel-resistant breast cancer cells. Furthermore, our previous studies have also demonstrated that DNMT3B can inhibit LATS2 expression through DNA methylation, thereby promoting YAP1 nuclear translocation and enhancing tumor cell proliferation while suppressing cell death, which may represent another regulatory mechanism for the reduced ferroptosis observed in paclitaxel-resistant breast cancer (Wu et al., 2019). We will further explore the relevant mechanisms from this perspective in our subsequent research.

Ferroptosis is an iron-dependent, non-apoptotic form of regulated cell death characterized by the biochemical hallmarks of intracellular iron accumulation, massive lipid peroxide (LPO) buildup, and dysfunction of antioxidant systems (particularly GPX4), ultimately leading to cell membrane structural damage and cell death (Jiang et al., 2021). Our study revealed that ferroptosis is significantly suppressed in paclitaxel-resistant TNBC, which aroused our great interest. Furthermore, among ferroptosis-related proteins, we found that SLC25A6 expression was markedly reduced, and this downregulation is significantly associated with DNA methylation by DNMT3B. SLC25A6 regulates ferroptosis by modulating the opening of the mitochondrial permeability transition pore (mPTP), influencing mitochondrial energy metabolism and oxidative stress, serving as a critical node in mitochondria-mediated ferroptosis (Lu et al., 2025). It interacts with cyclophilin D and other proteins to constitute the mPTP, thereby regulating mitochondrial membrane permeability, mitochondrial ROS generation, and the initiation of ferroptosis (Wang et al., 2023). Studies have shown that ferroptosis inducers (Erastin and RSL3) can activate mPTP opening, and as a key component of the mPTP, SLC25A6 expression directly affects the opening threshold (Lu et al., 2025). Furthermore, high functional expression of SLC25A6 leads to impaired ADP/ATP transport, disruption of mitochondrial respiratory chain function, and massive generation of mitochondrial ROS, providing oxidants for lipid peroxidation and thereby accelerating ferroptosis (Liu et al., 2025). In ovarian cancer, MRPL13 can promote K48-linked ubiquitination and degradation of SLC25A6, thereby inhibiting mPTP opening, reducing mtROS and cytochrome c release, conferring resistance to ferroptosis, and facilitating tumor survival and progression (Liu et al., 2025). Multiple studies have demonstrated that downregulation of SLC25A6 enhances mitochondrial function and suppresses cell death, representing a potential mechanism underlying tumor drug resistance, whereas targeting SLC25A6 can restore the sensitivity of tumor cells to ferroptosis inducers (Clémençon et al., 2013). Our findings are consistent with the aforementioned results. More importantly, DNMT3B regulates the expression of SLC25A6 protein by methylating the 252 CpG island site in its DNA through its DNA methylation function. Since multiple studies have shown that SLC25A6 significantly modulates tumor ferroptosis, our research also confirms that the DNMT3B-SLC25A6 regulatory axis can significantly affect the expression of key proteins involved in ferroptosis and markedly modulates ferroptosis in paclitaxel-resistant TNBC cells.

Of course, our research still has some limitations, such as why DNMT3B was chosen instead of TK1 and IL-5 for epigenetic studies. First, although TK1 is widely recognized as a proliferation marker in various malignancies, its clinical application in breast cancer is primarily limited to monitoring treatment response and detecting residual disease, rather than serving as a key mechanistic driver of chemotherapy resistance (Zhou et al., 2012). TK1 reflects the cellular proliferative state but does not directly participate in the epigenetic reprogramming upon which acquired drug resistance in triple-negative breast cancer (TNBC) depends (Zhou et al., 2012). Second, regarding IL-5, literature review and preliminary data demonstrate that there is no significant difference in serum IL-5 levels between breast cancer patients and benign breast tumor controls (P = 0.857), and IL-5 expression shows no correlation with TNM stage, lymph node metastasis, or molecular subtypes (including TNBC) (Raju et al., 2025). Furthermore, in multivariate analysis, IL-5 was not identified as an independent risk factor for breast cancer malignancy, whereas IL-6, IL-17, and VEGF exhibited significant correlations (Raju et al., 2025). These findings suggest that IL-5 may not be a critical mediator of paclitaxel resistance in TNBC, although it may participate in eosinophil-mediated anti-tumor immunity in the context of immunotherapy (Grisaru-Tal et al., 2022). However, whether TK1 and IL-5 contribute to chemotherapeutic resistance in TNBC through alternative mechanisms or modes of action remains a direction for our future research.

In summary, our study demonstrates that high expression of DNMT3B in paclitaxel-resistant triple-negative breast cancer suppresses the expression of SLC25A6 by promoting DNA methylation of its 252-bp CpG island, which significantly inhibits ferroptosis in paclitaxel-resistant TNBC cells and accelerates their malignant biological progression.

## Data Availability

The datasets presented in this study can be found in online repositories. The names of the repository/repositories and accession number(s) can be found in the article/Supplementary Material.

## Supplementary material

SUPPLEMENTARY FIGURE S1
**(A)** Effect of paclitaxel on proliferation in MDA-MB-231 and Hs578T. **(B)** Effect of paclitaxel on clone formation in MDA-MB-231 and Hs578T. **(C)** Effect of paclitaxel on apoptosis in MDA-MB-231 and Hs578T. **(D)** Effect of paclitaxel on invasion and migration in MDA-MB-231 and Hs578T. ***p < 0.01*.

SUPPLEMENTARY FIGURE S2
**(A)** Effect of paclitaxel on mitochondrial morphology in MDA-MB-231 and Hs578T. **(B)** Effect of paclitaxel on MDA in MDA-MB-231 and Hs578T. **(C)** Effect of paclitaxel on iron ion in MDA-MB-231 and Hs578T. **(D)** Effect of paclitaxel on lipid peroxidation in MDA-MB-231 and Hs578T. **(E)** Effect of paclitaxel on ROS in MDA-MB-231 and Hs578T S. **(F)** Effect of paclitaxel on Fe^2+^ MDA-MB-231 and Hs578T.***p < 0.01*.

SUPPLEMENTARY FIGURE S3
**(A)** Effect of Ferrostatin-1 on MDA in MDA-MB-231-PTXS and Hs578T-PTXS. **(B)** Effect of Ferrostatin-1 on iron ion in MDA-MB-231-PTXS and Hs578T-PTXS. **(C)** Effect of Ferrostatin-1 on lipid peroxidation in MDA-MB-231-PTXS and Hs578T-PTXS. **(D)** Effect of Ferrostatin-1 on ROS in MDA-MB-231-PTXS and Hs578T-PTXS. **(E)** Effect of Ferrostatin-1 on Fe^2+^ MDA-MB-231-PTXS and Hs578T-PTXS.**p < 0.05*.

SUPPLEMENTARY FIGURE S4
**(A)** Effect of 5-Aza-2 ′- deoxycytidine on proliferation in MDA-MB-231-PTXS and Hs578T-PTXS. **(B)** Effect of 5-Aza-2 ′- deoxycytidine on clone formation in M MDA-MB-231-PTXS and Hs578T-PTXS. **(C)** Effect of5-Aza-2 ′- deoxycytidine on MDA in MDA-MB-231-PTXS and Hs578T-PTXS. **(D)** Effect of 5-Aza-2 ′- deoxycytidine on iron ion in MDA-MB-231-PTXS and Hs578T-PTXS. **(E)** Effect of 5-Aza-2 ′- deoxycytidine on lipid peroxidation in MDA-MB-231-PTXS and Hs578T-PTXS. **(F)** Effect of 5-Aza-2 ′- deoxycytidine on ROS in MDA-MB-231-PTXS and Hs578T-PTXS. **(J)** Effect of 5-Aza-2 ′- deoxycytidine on Fe^2+^ MDA-MB-231-PTXS and Hs578T-PTXS. **p < 0.05;**p < 0.01*.

SUPPLEMENTARY FIGURE S5
**(A)** CCK8 analysis of cell proliferation in paclitaxel-resistant TNBC/Si-DNMT3B after over-expression of SLC25A6. **(B)** Clone formation in paclitaxel-resistant TNBC/Si-DNMT3B after over -expression of SLC25A6. **(C)** MDA assay analysis of malondialdehyde in paclitaxel-resistant TNBC/Si-DNMT3B after over-expression of SLC25A6. **(D)** Iron ion assay analysis of iron ion in in paclitaxel-resistant TNBC/Si-DNMT3B after over-expression of SLC25A6. **(E)** Lipid peroxidation assay analysis of lipid peroxidation in paclitaxel-resistant TNBC/Si-DNMT3B after over-expression of SLC25A6. **(F)** Lipid ROS assay analysis of ROS in paclitaxel-resistant TNBC/Si-DNMT3B after over-expression of SLC25A6. **(J)** Immunofluorescence analysis of Fe^2+^ in paclitaxel-resistant TNBC/Si-DNMT3B after over-expression of SLC25A6. **(H)** *In vivo* animal model analysis of tumorigenesis in in paclitaxel-resistant TNBC/Si-DNMT3B after over-expression of SLC25A6. **p < 0.05*.
